# The Role of Cell and Gene Therapies in the Treatment of Infertility in Patients with Thyroid Autoimmunity

**DOI:** 10.1155/2022/4842316

**Published:** 2022-08-30

**Authors:** Sanja Medenica, Dzihan Abazovic, Aleksandar Ljubić, Jovana Vukovic, Aleksa Begovic, Gaspare Cucinella, Simona Zaami, Giuseppe Gullo

**Affiliations:** ^1^Department of Endocrinology, Internal Medicine Clinic, Clinical Center of Montenegro, School of Medicine, University of Montenegro, Podgorica, Montenegro; ^2^Biocell Hospital, Belgrade, Serbia; ^3^Special Gynecology Hospital with Maternity Ward Jevremova, Belgrade, Serbia; ^4^Libertas International University, Dubrovnik, Croatia; ^5^Department of Obstetrics and Gynecology, Villa Sofia Cervello Hospital, IVF UNIT, University of Palermo, Palermo, Italy; ^6^Department of Anatomical, Histological, Forensic and Orthopedic Sciences, “Sapienza” University of Rome, Rome, Italy

## Abstract

There is a rising incidence of infertility worldwide, and many couples experience difficulties conceiving nowadays. Thyroid autoimmunity (TAI) is recognized as one of the major female infertility causes related to a diminished ovarian reserve and potentially impaired oocyte maturation and embryo development, causing adverse pregnancy outcomes. Growing evidence has highlighted its impact on spontaneously achieved pregnancy and pregnancy achieved by in vitro fertilization. Despite the influence of thyroid hormones on the male reproductive system, there is insufficient data on the association between TAI and male infertility. In past years, significant progress has been achieved in cell and gene therapies as emerging treatment options for infertility. Cell therapies utilize living cells to restore healthy tissue microenvironment and homeostasis and usually involve platelet-rich plasma and various stem cells. Using stem cells as therapeutic agents has many advantages, including simple sampling, abundant sources, poor immunogenicity, and elimination of ethical concerns. Mesenchymal Stem Cells (MSCs) represent a heterogeneous fraction of self-renewal, multipotent non-hematopoietic stem cells that display profound immunomodulatory and immunosuppressive features and promising therapeutic effects. Infertility has a genetic component in about half of all cases, although most of its genetic causes are still unknown. Hence, it is essential to identify genes involved in meiosis, DNA repair, ovarian development, steroidogenesis, and folliculogenesis, as well as those involved in spermatogenesis in order to develop potential gene therapies for infertility. Despite advances in therapy approaches such as biological agents, autoimmune disorders remain impossible to cure. Recent research demonstrates the remarkable therapeutic effectiveness of MSCs in a wide array of autoimmune diseases. TAI is one of many autoimmune disorders that can benefit from the use of MSCs, which can be derived from bone marrow and adipose tissue. Cell and gene therapies hold great potential for treating autoimmune conditions, although further research is still needed.

## 1. Introduction

**Figure 1:**
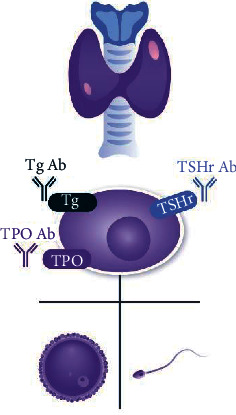
The negative effect of thyroid autoimmunity on female and male fertility.

Infertility is recognized as a growing issue worldwide, affecting about 8–12% of reproductive-age couples [1]. In order to establish and maintain pregnancy, the endocrine and immune systems must work in harmony to sustain normal functioning while also adapting to novel conditions. As a result, endocrine and immune system disorders, or a combination of the two, could cause infertility and reproductive dysfunction. Thyroid autoimmunity (TAI) is the most prevalent autoimmune disorder in women of reproductive age, with an estimated prevalence of 5–15% [2]. It is characterized by the presence of anti-thyroid peroxidase antibodies (TPO-Abs) and/or anti-thyroglobulin antibodies (Tg-Abs), with antibody-dependent cell-mediated cytotoxicity against human thyroid cells [3]. Rising values of TPO-Abs and TG-Abs are the hallmark of Hashimoto's thyroiditis which can lead to hypothyroidism. The increase of stimulating TSHR-Abs is the hallmark of Graves' disease, resulting in hyperthyroidism. Those two conditions can be seen as the two opposing ends of a continuous variety of TAI [4]. Women diagnosed with TAI have an increased risk of developing subclinical and further overt hypothyroidism, extensively known for its negative impact on fertility, spontaneous and *in vitro* fertilization (IVF) achieved pregnancy outcomes, and child neurodevelopment. However, there is a growing body of evidence that TAI per se may influence reproductive health, independently of thyroid hormone levels [5]. It is hypothesized that the thyroid autoantibodies, through various immunological mechanisms, may disturb the normal process of folliculogenesis, fertilization, implantation, and even post-implantation embryo development leading to adverse pregnancy outcomes conceived either spontaneously or via assisted reproductive technologies [6] (Figure 1).

TPO Ab-thyroid peroxidase antibody, Tg Ab-Thyreoglobulin antibody, TSH Ab- thyroid stimulating hormone antibody, TPOr-thyroid peroxidase ,Tgr-thyreoglobulin , TSHr- thyroid stimulate hormone receptor.

TAI has been reported more frequently in women with decreased ovarian reserve, ovarian dysfunction, endometriosis, as well as in women with unexplained infertility and implantation failure [7–9]. Autoimmunity and inflammation have been linked to both polycystic ovary syndrome (PCOS) and endometriosis, and those conditions are often present with high prevalence in women with autoimmune thyroid disease struggling with infertility [8, 10, 11]. The impact of TPO-Ab on spermatogenesis is still not clear [12].

The field of cell and gene therapies is one of the most rapidly evolving. These therapies hold the potential to treat conditions previously thought untreatable and apply to a wide range of diseases. These therapies are the future of personalized medicine.

In this review, we summarize the relationship between TAI and fertility and the potential of state-of-the-art cell and gene therapies for managing TAI-related infertility. We focus on the therapies that have already been approved and have shown promising effects, such as mesenchymal stem cells (MSC) and platelet-rich plasma (PRP). Additionally, we report the potential of subcellular therapies using secretomes, secreted biomolecules from therapeutic cells. We discuss future therapeutic prospects such as Chimeric antigen receptor T cells (CAR T) therapies and gene therapy with various targets. Also, we consider the potential of MSC therapy for treating TAI and thyroid dysfunction effect on male infertility. Finally, we discuss the limitations and obstacles to implementing these advanced yet experimental treatment strategies in clinical practice settings.

## 2. Material and Methods

In order to conduct this narrative review, we searched PubMed and Google Scholar for all studies published up to March 2022. The keyword search used included “thyroid and female fertility,” “thyroid and pregnancy”, “hypothyroidism and pregnancy,” “thyroid autoimmunity and pregnancy,” “thyroid autoimmunity and assisted reproductive technologies,” “thyroid and recurrent pregnancy loss”, “thyroid and IVF,” “PRP and ovarian failure,” “PRP and thin endometrium,” “stem cells and female fertility,” “senescent cells and female fertility,” “senescent cells and elimination,” “gene therapy and premature ovarian insufficiency (POI),” “thyroid and infertile males,” “intracytoplasmic sperm injection (ICSI) treatment,” “MSC and autoimmune diseases.” Exclusion criteria: articles written in a non-English language or not relevant to the present review.

### 2.1. Thyroid Autoimmunity and Female Fertility

The follicular fluid contains a complex combination of proteins, nucleic acids, and other metabolites and molecules, which indicate the metabolic activity of the follicle. Oocyte and embryo quality and subfertility have been associated with various molecules found in the follicular fluid [13]. The fluid milieu serves as an essential microenvironment for regulating the development and quality of the oocytes. The neighbors' cells can communicate through the follicular fluid by secreting various signaling molecules in extracellular vesicles [13, 14]. More important for our review, thyroid hormones and enzymes necessary for their synthesis have been discovered in follicular fluid [15, 16]. A pioneering study conducted by Monteleone et al. has revealed the presence of TPO-Abs in follicular fluid [16]. Perifollicular blood vessel network formation enables nutrients and growth factors to enter the developing follicle. TPO antibodies are likely to cross the “follicle-blood” barrier during the maturation period and reach concentrations proportional to their blood concentrations. One proposed hypothesis of mediated infertility is that TPO-Abs may damage the ovarian follicles via antibody-mediated cytotoxicity, while another is based on structural antigen similarity between zona pellucida and thyroid tissue, which may lead to cross-reactivity of TPO-Ab toward zona pellucida [16].

POI is linked to various autoimmune diseases, most commonly thyroid disorders [17]. Women with TAI have an increased risk of developing POI, however, the pathophysiology effect on ovarian reserve has not been well understood [18]. Two studies have investigated the correlation between the ovarian reserve and TPO-Abs positivity in women seeking infertility treatment. One study, including only patients diagnosed with idiopathic infertility, reported a significant association between the idiopathic low ovarian reserve and TPO-Abs presence, whereas the second study described lower antral follicular count in TPO-Abs positive women with a decreased ovarian reserve and unexplained infertility [19, 20]. Moreover, due to the presence in the ovarian follicles, TPO Abs has the potential to stimulate the immune system, which may damage ovarian tissue [18].

### 2.2. Thyroid Autoimmunity and Spontaneous Pregnancy

Scientific findings highlight the relationship between pregnancy loss and TAI. It has been pointed out that patients with positive thyroid autoantibodies have a higher miscarriage rate than thyroid autoantibody negative patients [21], with more than two-fold odd ratio (OR) in TPO-Abs positive women [22]. In the meta-analysis including 31 studies evaluating miscarriage, 28 showed a positive association between thyroid autoantibodies and miscarriage, the cohort studies showed more than tripling in OR of miscarriage and incase-control studies OR for miscarriage was 1.80. [23]. Confirming similar results, Chen and Hu revealed that women with TAI were found to have slightly higher age and TSH levels [24].

Additionally, an association between recurrent pregnancy loss and TAI has been demonstrated [25]. The suggested pathophysiological mechanism of miscarriage involves immunological dysfunction, particularly over-activity of Th1-cell response that may induce an inflammatory reaction and lead to miscarriage either via T cells or NK cells [26]. Increased numbers of CD5 and CD20 + B lymphocytes have also been measured in women with recurrent miscarriages [27].

Preterm delivery is the next most investigated unfavorable pregnancy outcome in women diagnosed with TAI. Previous studies have emphasized significantly increased relative risk or odds ratio risk for preterm birth in TPO-Abs positive women [23, 28–30].

Another less frequently reported effect besides infertility, which is the centerpiece of this review, is a higher rate of adverse maternal outcomes in patients with TAI, such as increase in the risk of placental abruption [31]. Higher perinatal mortality in TPO-Ab positive women, regardless of their hormonal status, has also been reported [32]. Results regarding the effect of the supplementation with levothyroxine (LT4) on reducing the risk of pregnancy loss and premature birth in women with TAI appear to be incoherent [23, 25, 33]. Moreover, some authors have investigated the TSH levels in women diagnosed with subclinical hypothyroidism in euthyroid patients on the risk of miscarriage [22, 24]. Although the value of TSH levels in pregnancy remains controversial, concerning the documented increase in TSH levels throughout the gestation, American Thyroid Association (ATA) recommends measuring TSH in euthyroid, TPO-Ab positive women, at the time of pregnancy confirmation and every 4 weeks during the mid-pregnancy [34].

### 2.3. Thyroid Autoimmunity and Assisted Reproductive Technologies

A large number of existing studies in the broader literature have examined the nature of the relationship between TAI and Assisted Reproductive Technologies (ARTs) outcomes, although the results have remained debatable. Two meta-analyses have reported an increased rate of miscarriage and decreased live birth rate in euthyroid, TAI-positive women who underwent IVF and/or ICSI treatment compared to controls [35, 36]. A considerable number of meta-analyses have not revealed a discrepancy, nor in miscarriage rate or clinical pregnancy rate between the same groups of women [37–40].

Supplementation with LT4 in TPO-Ab positive euthyroid women going through IVF did not show improvements in decreased miscarriage rate or increased clinical pregnancy rate [41]. Moreover, another study reported the absence of a diminution in pregnancy loss rate or preterm birth rate in TAI women receiving LT4 in advance of IVF procedure. In addition, the same study noticed that the results were opposite in patients who conceived naturally [33].

Heterogeneity and inconsistency of results do not offer any straightforward conclusions. Nevertheless, it may be essential to consider the following points. First, the study done by Poppe et al. focused solely on the ICSI procedure [40]. Concerning the previously given thesis of TPO-Ab cross-reactivity toward the zona pellucida antigens, and subsequent disabling of the normal fertilization process, which may be avoided by direct spermatozoa injection, it appears reasonable that LT4 supplementation may not exert any positive effects. This may not be the case with IVF. However, all of the other studies have included different ART procedures, mainly IVF and ICSI, which may be the cause of negative results. Therefore, future clinical trials should concentrate on one specific ART protocol to obtain more precise results. Additionally, there has been observed a disparity between the outcomes of naturally conceived versus ART pregnancies in TAI-positive women whether or not they have received LT4 supplementation. These results imply that future research should evaluate these pregnancies separately.

Finally, there is an ongoing debate concerning adequate TSH serum levels in women undergoing ART treatment. A few studies have examined TSH concentrations in TAI-positive women prior to IVF or ICSI procedure, but they appear to be inconsistently as well [35, 37]. Due to the well-documented effects of thyroid dysfunction on reproductive function in general, the European Thyroid Association (ETA) recommends mandatory screening for TSH and TAI in all women prior to infertility treatment. If the TSH levels appear to be >4 mIU/l, LT4 supplementation is obligatory. In women with TAI and TSH >2.5 mIU/l and <4.0 mIU/L alow dose of LT4 was suggested before ovarian stimulation on a case-by-case basis, with respect to previous recurrent miscarriages, age >35 years, and ovarian causes of subfertility, so the decision on LT4 treatment should be estimated individually [42].

### 2.4. Biological Therapies for TAI-Related Infertility

#### 2.4.1. Intraovarian and Endometrial PRP Application

One of the growing and emerging trends in infertility therapies is the use of PRP. Several recently published studies confirmed the positive effects of PRP in reproductive medicine. PRP, for example, promotes the growth of isolated human primordial and primary follicles to the preantral stage [43]. In addition, intraovarian injection of PRP improves ovarian reserve markers and hormonal dysfunction in patients with POI, premenopausal women, and low responders to IVF stimulation, allowing some previously infertile patients to conceive and give birth [44–46]. The therapeutic effect of PRP has also been shown in increasing endometrium thickness in patients with refractory thin endometrium [47–50].

PRP is a biological product that contains at least four times the number of platelets found in peripheral blood and has been used in different fields of medicine for tissue repair and regeneration [51]. PRP is made from a patient's blood, therefore, it is considered relatively safe and poses no risk of tissue rejection or inflammatory reactions. It contains growth factors such as platelet-derived growth factor (PDGF), transforming growth factor-beta (TGF-b), fibroblast growth factor (FGF), epidermal growth factor (EGF), vascular endothelial growth factor (VEGF), etc. Hormones, cytokines, and chemokines are also included [52]. These molecules play a role in various cellular processes, including anabolic signaling, cell proliferation, differentiation, migration, angiogenesis, and apoptosis, by inducing the transcription of genes involved in these processes [53, 54].

Regarding TAI, these biomolecules play a critical role in ovarian function. They have a dynamic role in oocyte and follicle growth and maturation, regulating ovarian tissue regeneration and enhancing the microenvironment [55]. Further, patients with thyroid dysfunction could have prolonged clotting and bleeding time, thus it is advisable to control these factors when using PRP [56]. It is known that prothrombotic states share similar immunological features with TAI, and the role of thrombophilia associated with TAI in unexplained infertility, implantation failure, and recurrent miscarriage should not be neglected [7]. In addition, we may anticipate that the efficiency of PRP treatments will be lowered in hyperthyroidism, while it should not be affected in hypothyroidism [57]. Thus, precise quality control through platelet count and growth factor content should be determined when producing PRP, and the state of euthyroidism before the procedure must be provided.

#### 2.4.2. Stem Cells

Undifferentiated cells with the potential to renew themselves for long periods without significant changes in their general features are known as stem cells. Embryonic and induced pluripotent stem cells have limits in the clinical practice application, thus, there has been a lot of interest in MSCs, which are free of both ethical problems and the creation of teratomas [58].

Embryonic stem cells (ESCs), MSCs, stem cells from extra-embryonic tissues, induced pluripotent stem cells (iPSCs), spermatogonial stem cells, and ovarian stem cells are all applied in stem cell-based therapy for infertility [59].

MSCs are a type of stem cells that originates in the mesoderm. They can self-renew and differentiate into ectodermic and endodermic cells as well as mesoderm lineages, including chondrocytes, osteocytes, and adipocytes. MSCs can be harvested from several tissues, such as bone marrow, menstrual blood, adipose tissue, the umbilical cord, and the placenta. They are multipotent, widely available, poorly immunogenic, and therefore, they emerged as promising therapeutics [59, 60].

MSCs can move directly to the damaged ovary and survive there under the activation of numerous stimuli in the case of ovarian dysfunction and facilitating ovarian healing. According to published research, the amount of differentiated and functionally integrated MSCs is insufficient to explain observed ovarian function improvements. Furthermore, it is unknown whether MSCs develop into oocytes after moving to damaged tissue. Paracrine pathways may be to blame for the improved ovarian function seen in these investigations. These methods entail the production of cytokines, chemokines, micro RNAs, and growth factors, including VEGF, IGF, and HGF, which can aid ovarian repair by promoting angiogenesis, anti-inflammation, immunoregulation, anti-apoptosis, and anti-fibrosis. More research is needed to provide information if MSCs can differentiate into oocytes or supportive cells, which can improve ovarian function and eventually treat ovarian dysfunction. MSC transplantation as a clinical therapy would benefit from this differentiation as well [60].

Bone marrow MSCs (BMSCs) have been shown to enhance neovascularization, minimize granulosa cell apoptosis, and improve physiological oocyte maturation [61]. In animal models of ovarian failure, injections of isolated BMSCs directly into the ovarian tissue or via an artery have been used extensively in the successful restoration of ovarian function; however, there are currently very few investigations in humans [62, 63]. According to our knowledge, there have been several case reports of autologous BMSCs used in the clinical treatment of women with ovarian failure that resulted in successful births [64–66]. In animal models, the therapeutic effect of MSCs has also been shown through hormonal status improvement, restoration of folliculogenesis, and improvement of the ovarian microenvironment. Namely, in a rabbit model of premature ovarian failure (POF), MSCs enhanced ovarian reserve and follicular number and improved hormonal state [67]. A similar has been observed in human mesenchymal stem cells (hMSCs) to treat POF in a mouse [68]. Moreover, human menstrual blood-derived stem cells have also shown benefits in multiple mouse studies by improving the ovarian microenvironment and hormonal state [68–70].

Based on the findings of the previous investigations, Ljubić et al. developed and tested a method that included ovarian cortex laparoscopic biopsy, stem cell therapy, growth factor incubation, and *in vitro* activation—SEGOVA rejuvenation program, which has been described thoroughly in Tinjić et al. [71].

#### 2.4.3. MSC Secretome

Numerous studies have shown that paracrine substances, different proteins, signaling lipids, and nucleic acids [72] generated by MSCs are responsible for immunomodulation, proliferation and differentiation promotion, apoptosis inhibition, and other healing effects in injured tissues [73]. Bioactive substances can be released free or packaged in extracellular vesicles called exosomes. They act as shuttles for certain genetic information, proteins, and messenger RNA to other cells. They allow cell-to-cell communication, transporting molecules that are important regulators of intracellular information between close and distant cells [73, 74]. Exosomes (produced from amniotic fluid stem cells—AFSCs) prevented atresia of ovarian follicles in POI-induced mice, where antiapoptotic effects on granulosa cells injured by chemotherapy *in vitro* were observed [75]. Others also reported favorable effects of MSCs secretome on ovarian function in animal models—decreased apoptosis of oocytes and granulosa cells [67, 75], the increased proliferation of human granulosa cells *in vitro,* and the expression of enzymes involved in the estrogen production [76].

#### 2.4.4. Gene Therapy

POI is characterized by the loss of normal ovarian function before the age of 40 years, described by deficient ovarian sex hormones and diminished ovarian reserve [77]. There are a number of health issues associated with POI, including menopausal symptoms (such as night sweats, hot flashes, and vaginal dryness), infertility, and an increased rate of autoimmune disorders [78]. Hormone replacement therapy, which is the primary therapeutic option for POI, can help relieve the symptoms of the condition. Only a minority of women with POI and a normal karyotype will recover spontaneously, with a fertility rate of 5%. Due to ineffective treatment for POI-related infertility, egg donation is often the patient's only choice for having biological children, with all the clinical, legal, and ethical implications that entail [79].

POI has been characterized as mainly idiopathic, with an underlying genetic cause in about 20–25% [80]. Because of breakthroughs in genetics and next-generation sequencing, most idiopathic forms will probably show a genetic etiology [81]. Several genes are being investigated as possible therapeutic targets for POI women, such as FSH receptor (FSHR), SALL4, Sphingomyelin phosphodiesterase 1 (SPD1), Neonatal Thymulin Gene, Basonuclin-1. The genes mentioned above may be good candidates for gene therapy in order to correct mutations that contribute to POI [82].

The revolutionary aspect of gene therapy is its ability to cure the disease completely. Unlike continuous administration of medications with a short half-life, continuous gene expression could provide a cure with a single treatment. An end-to-lifetime treatment instead of a permanent cure represents a paradigm shift in modern medicine. Gene therapy was first developed for treating monogenic diseases [83], with the idea to transfer genetic material to restore or modify target cells' function by introducing a new gene, modifying existing or downregulating a specific gene of interest. Adding a new, functional copy of the gene would enable the supply of the protein, otherwise missing due to a particular mutation. Owing to the remarkably rapid development and improvement of genetic editing technology, the gene of interest can also be corrected depending on the mutation that is causing the problem. The discovery of RNA interference and small non-coding RNAs has led to several other innovative approaches for gene silencing, including miRNA binding to mRNA to downregulate the expression of a specific gene [84]. The delivery of the genetic material to a specific location remains one of the significant challenges in the field of gene therapy. One of the most promising genes therapy delivery techniques is via viral vectors [85]. However, viral vectors bring several issues, including the limited DNA size they can carry and immune reaction. Non-viral vectors investigated so far, on the other hand, have not shown promising results [83, 84]. The most promising method and probably the future of this therapy would be a combination of stem cell therapy and gene transfer. Patients' stem cells would be harvested, the target gene would be modified *in vitro*, and the cells would be transplanted back into the patient. In this approach, a viral vector would not be required, and the resulting stem cells could provide rise to a new pool of cells with the required function [81, 83].

#### 2.4.5. CAR T Therapy for Eliminating Senescent Cells

Cellular senescence is an irreversible growth arrest when cells are exposed to various cellular stresses. There are numerous intrinsic and extrinsic factors, including telomere shortening and structure changes, radiation, oxidative and genotoxic stress, epigenetic changes, inflammation and/or tissue damage signals, as well as developmental signals, that can cause cells to enter a stable cell cycle arrest [86]. Biologically, this process serves to keep damaged cells from proliferating. However, these cells retain their synthetic activity, coupled with dysfunctional intracellular processes that alter their secretory profile, known as senescence-associated secretory phenotypes (SASP). SASP includes a variety of signaling molecules, including pro-inflammatory cytokines, chemokines, and proteases, which have been demonstrated to be able to spread senescence in nearby cells [86, 87]. Some of the molecules produced by the SASP could explain how senescent cells can alter the tissue microenvironment and draw in the immune system through autocrine and paracrine activity and, paradoxically to its biological role, cause malignant phenotypes [87, 88].

Endometriosis is a common gynecological inflammatory condition characterized by the development of functional endometrial-type mucosa outside the uterine cavity [89]. It is estimated that endometriosis affects about 10% of women of reproductive age and up to 50% of infertile women [90]. The pathogenesis of endometriosis is still largely unknown however it appears to be linked to various immune cell types [91]. There have been various common mechanisms between endometriosis and autoimmune disorders, including abnormal functions of T and B cells, polyclonal B cell activation, and inflammatory tissue damage [9, 91]. A growing number of evidence support the theory of the involvement of immune cells in the pathogenesis of endometriosis, thus, regulating the actions of these cells may modulate the onset or progression of the disease. Granulosa cells' senescence has been proved in endometriosis-associated infertility [92]. Furthermore, the accumulation of senescent cells creates an inflammatory milieu that can lead to chronic tissue damage and can contribute to various diseases [92, 93]. It is possible that such an event could occur in the ovary, contributing to infertility. Considering all this, finding a way to eliminate senescent cells could potentially improve the tissue microenvironment by reducing tissue inflammation and organ dysfunction, which might also reduce the risk of cancer [94]. Therefore, eliminating already-formed senescent cells might increase the chance of pregnancy.

Promising cell therapy to eliminate senescent cells that is currently under investigation is CAR T therapy. Amor et al. demonstrated that the urokinase-type plasminogen activator receptor (uPAR) could be used as a specific cell-surfaced marker during cell senescence, which could be used as the target for chimeric antigen receptors [95]. They have shown that uPAR protein is highly expressed on the surface of senescent cells and provide proof-of-principle of the therapeutic potential of CAR T cells in removing senescent cells *in vitro* and *in vivo*. However, additional clinical studies are required to demonstrate the safety and dose of such therapy while minimizing toxicity.

### 2.5. Thyroid Autoimmunity and Male Infertility

The prevalence of TAI in infertile men was estimated to be 7.5%, and the increased titers of ATA (anti-thyroid antibodies) were associated with semen defects such as asthenospermia and pathozoospermia [12]. TAI as the cause of male infertility has received little attention, and literature concerning this field is very scarce.

For the purpose of this review, Hashimoto's thyroiditis and Graves' disease as manifestations of TAI will be used to describe their effects on male infertility.

Only a few studies have investigated the effects of hyperthyroidism on semen quality. In 1992, Hudson and Edwards evaluated testicular function in 16 thyrotoxic patients [96]. They found the mean sperm densities did not significantly differ from controls. In the study conducted by Abalovich et al., the effect of hyperthyroidism on spermatogenesis was assessed in 21 patients; nine patients (43%) had a decreased total sperm count, 18 (85.7%) had “grade a”-linear motility defects, and 13 (61.9%) showed “progressive motility” problems [97]. Previous research done by Krassas et al. has compared the thyrotoxic patients to healthy controls via two semen analyses before and five months after achieving euthyroid state, which revealed a normal range of mean semen volume in both groups. However, mean sperm motility was significantly decreased in comparison to healthy controls. Five months following the treatment, thyrotoxicosis ameliorated sperm density and motility, with no effects on morphology. The patients' group showed lower mean sperm density, as well as morphology, but without statistical significance [98].

Hypothyroidism is associated with decreased sexual desire, impotence, and delayed ejaculation [99]. However, there is limited data concerning the effects of hypothyroidism on spermatogenesis and fertility of men [100]. Variations in testis volume and histology have been found in autopsy material [101, 102]. Histologically, in patients with prepubertal onset of hypothyroidism, there were abnormalities such as “delayed maturation,” and “involution of adult characteristics,” which were present in tubular content, tubular wall, and intertubular connective tissue. In cases of after-puberty hypothyroidism histological changes of the testes were seen only in the tubular content and presented as “involution” [101]. Short-term hypothyroidism after puberty does not alter semen quality sufficiently enough to impair male fertility [103, 104]. Semen density, motility, and morphology were not affected by subclinical hypothyroidism, as shown by Trumer et al. [12].

A bigger picture concerning male infertility and TAI cannot be seen, and conclusions cannot be made with such an enormous lack of research and knowledge on this subject. Thus, further research is needed to explore possible therapeutic options for these patients.

### 2.6. Cell Therapy for TAI

With the advancement in developing novel treatment modalities, such as biological agents, it is still not possible to cure autoimmune diseases. Cell therapy is a contemporary approach for curing severe diseases, such as autoimmune, that have shown great potential. MSCs display profound immunomodulatory and immunosuppressive features and promising therapeutic effects [105]. Recent studies demonstrate the remarkable therapeutic effectiveness of MSCs toward a wide array of autoimmune diseases such as Chron disease, multiple sclerosis, rheumatoid arthritis, systemic lupus erythematosus, and others [106]. As one of many autoimmune diseases, thyroid autoimmune disorders are targeted for the appliance of MSCs, derived from bone marrow and adipose tissue, as well as gene therapy.

An animal study conducted by Ma et al. on a mice model of Hashimoto's thyroiditis suggests that ICAM-1-expressing MSCs have the ability to ameliorate the expression of thyroxine (T4), TSH, TPO-Abs, and TgAbs *in vivo*, to diminish thyroid follicle injury and to lower splenic index in mice. ICAM-1/MSCs enhanced mRNA expression of anti-inflammatory cytokines, they modulated spleen signaling pathways ERK and P38, and overexpression of ICAM-1 modulated nesting of MSCs, bringing them to the thyroid injury site [107].

A potentially promising approach to the cell-based treatment of Grave's disease, as one of the two most frequent thyroid autoimmune disorders, is the usage of T regulatory cells (Tregs) in suppressing unwanted immunological responses. Tregs are subset of T-lymphocytes with potent suppressive activity in destructive immune responses, and the adaptation of the maternal immune response to the semi-allogeneic fetus is necessary for pregnancy success, thus fetal rejection leading to miscarriage could be the consequence of maternal immunological intolerance [108]. To prevent these adverse outcomes, the plan would be to collect T helper cells from patients and train them to recognize the autoantigen, TSHr in this case, and re-administer them, expecting an immunosuppression effect against the TSHr [109].

## 3. Conclusion

In the time of advanced technologies and modern therapy approaches, infertility still stands as a burning and unattainable problem. TAI is a serious immunological aspect of infertility. Cell and gene therapy offer many possibilities for the treatment of what is thought to be an incurable disease. Even though these state-of-art cells and gene therapies hold tremendous promise and show significant potential, additional *in vitro* and clinical studies are required before definitive guidelines can be established for effective treatments.

## Data Availability

The datasets used and/or analyzed during the current study are available from the corresponding author on reasonable request.
